# Effects of Electric Light on the Eye

**Published:** 1881-05

**Authors:** 


### EFFECTS OF ELECTRIC LIGHT ON THE EYE.

An unexpected objection to this light arises from its effects on the eye. European observers state, that the frequent variations in intensity to which this light is subject give rise to sudden and frequently repeated changes in the pupil, and, consequently, in the accommodation, of the eye. A light of this kind, therefore, causes not only simple muscular fatigue of the eye, but also a considerable degree of blurring and indistinctness of the retinal image. The eye suffers both when the light is too dim and when it is too bright. In the former case, the object looked at must be brought close to the eye to be clearly seen, and an increaseed accommodative effort is thus called for, which in most instances results in myopia In the former case the simple intensity of the light produces undue contraction of the pupil, and consequently an increase in accommodative tension within the eye.

